# Increased Expression of Angiopoietin 2 and Tie2 in Rosacea

**DOI:** 10.3390/dermatopathology13010002

**Published:** 2025-12-25

**Authors:** Aysin Kaya, Jean-Hilaire Saurat, Nathalie Satta, Gürkan Kaya

**Affiliations:** 1 Department of Clinical Pharmacology and Toxicology, University of Geneva, 1211 Geneva, Switzerland; aysin.kaya@unige.ch (A.K.); jean.saurat@unige.ch (J.-H.S.); nathalie.satta@unige.ch (N.S.); 2 Departments of Dermatology and Clinical Pathology, University Hospital of Geneva, 4, Rue Gabrielle-Perret-Gentil, 1211 Geneva, Switzerland

**Keywords:** Angiopoietin 1, Angiopoietin 2, Tie2, pTie2, rosacea, immunohistochemistry

## Abstract

In this study we evaluated the expression of Angiopoietin 1, Angiopoietin 2, and Tie2 by immunohistochemistry in the skin of 10 patients with erythemato telangiectatic and papulopustular rosacea. Significantly increased expression of Tie2 and Angiopoietin 2 in the endothelial cells of the dermal vessels in rosacea skin vs. non-lesional skin (100% and 33.3% for Tie2, and 100% and 50% for Angiopoietin 2) was observed. There was no difference in the expression of Angiopoietin 1 and phosphorylated Tie2 (pTie2) between the lesional skin of rosacea and non-lesional skin.

## Simple Summary

Rosacea, a common skin disease causing facial redness and visible blood vessels, is believed to be linked to defective blood vessel control. In this study we found in rosacea skin elevated levels of the proteins Angiopoietin 2 and Tie2, which are involved in blood vessel growth and behavior. These findings suggest that the problem in rosacea might be an imbalance and dysfunction in existing vessel controls, leading to the unstable blood vessels seen in this disease.

## 1. Introduction

Rosacea is a chronic skin disease that affects millions of people worldwide, primarily characterized by persistent facial erythema, telangiectasia, and, in some cases, papules, pustules, and eye irritation [1]. It affects primarily the convex surfaces of the face. Clinically there are four subtypes described: erythematotelangiectatic, papulopustular, phymatous, and ocular [2]. Granulomatous variant also exists and is characterized by a peculiar clinical and histological presentation [3]. Rosacea is characterized by remissions and exacerbations and may have mild, moderate, and severe clinical forms. Although the primary cause of rosacea remains elusive, various factors such as genetics, immune system dysfunction, environmental triggers such as ultraviolet (UV) exposure, chemical and ingested agents, and microorganisms such as *Demodex* mites (a major contributor) have been implicated.

Histopathologically rosacea is characterized by a perivascular and perifollicular lymphohistiocytic inflammatory infiltrate containing occasional plasma cells, of which the intensity is higher in the papulopustular subtype than in the erythematotelangiectatic subtype. Papulopustular rosacea lesions may also show superficial folliculitis, subcorneal/intraepidermal collections of neutrophils realizing pustules and keratotic follicular plugging with no microcomedones, as seen in acne. Vascular changes (dilated vessels or telangiectasia and proliferation) and solar elastosis are seen in both subtypes, with no difference in frequency between the two groups. *Demodex* mites are observed in 20–50% of cases. The granulomatous subtype shows epithelioid granulomas in the vicinity of damaged hair follicles, sometimes with necrosis mimicking caseation and more mast cells than the erythematotelangiectatic form [4]. Phymatous rosacea shows sebaceous gland hypertrophy and scattered follicular plugging [5,6].

One significant aspect of rosacea, visible also histologically, is angiogenesis. Angiogenesis is a physiological process that involves the formation of new blood vessels from pre-existing ones. It plays a vital role in tissue repair, wound healing, and embryonic development. However, when angiogenesis becomes dysregulated, it can contribute to the pathogenesis of several diseases, including cancer, arthritis, and skin disorders such as rosacea.

In rosacea, angiogenesis is a central player in the development and progression of the condition. Several factors contribute to the abnormal angiogenesis seen in rosacea:

(i) Chronic inflammation: In response to various triggers, such as UV irradiation, microorganisms, and environmental factors, the immune system becomes overactive, leading to the release of inflammatory mediators. These mediators, such as interleukin-1 (IL-1) and tumor necrosis factor-alpha (TNF-α), promote angiogenesis by stimulating the production of proangiogenic factors. In addition, pattern recognition receptors, including Toll-like receptors (TLRs) and antimicrobial peptides (mainly cathelicidin in the skin), are activated.
(ii) Vascular Endothelial Growth Factor (VEGF): VEGF is a potent proangiogenic factor that plays a pivotal role in the formation of new blood vessels. Elevated levels of VEGF have been detected in the skin of individuals with rosacea. This increase in VEGF contributes to the enlargement and dilation of blood vessels, leading to the visible erythema associated with the condition.
(iii) The Angiopoietins and Tie2 signaling pathway: Angiopoietin 1 (Ang1, produced by the pericytes) and Angiopoietin 2 (Ang2, produced by the endothelial cells and stored in Weibel–Palade bodies) are proteins that competitively bind to the Tie2 receptor expressed on endothelial cells.

Our group recently focused on the Ang1&2/Tie2 signaling pathway, which is known as the keeper of vascular quiescence and had not been previously studied in rosacea patients [7].

In this study we explored the expression of Angiopoietin 1, Angiopoietin 2, and Tie2 by immunohistochemistry in rosacea lesions.

## 2. Subjects and Methods

Ten patients with erythematotelangiectatic or papulopustular rosacea attending the Dermatology Clinic of the University Hospital of Geneva were included in this study. Written informed consent of patients was obtained. All the subjects were treatment-free for at least 1 month prior to the skin biopsies. The study subjects included 5 female and 5 male patients (age range: 26–81 years, mean age: 55 years). Clinical classification and grading were performed according to the standard classification of rosacea [2]. Six age- and sex-matched non-lesional facial skin samples were also included as controls (three female and three male subjects, age range: 40–88 years, mean age: 56 years). The skin samples were fixed in 10% formalin and embedded in paraffin blocks for histopathological diagnostic purposes. The samples were cut into 5-µm sections and stained with anti-Angiopoietin 1 (rabbit polyclonal, 1:400, PA5-96841, Invitrogen, Carlsbad, CA, USA), Angiopoietin 2 (rabbit polyclonal, 1:400, PA5-118950, Invitrogen), Tie2 (goat polyclonal, 1:50, AF313, R&D Systems, Minneapolis, MN, USA), and pTie2 (rabbit polyclonal, 1:50, AF2720, R&D Systems) antibodies. Proper positive and negative controls were used in each run of the immunohistochemistry protocol. After staining, slides were scored using a scale of 0–3 according to the intensity of staining (0: no staining, 1+: slight staining, 2+: moderate staining, 3+: strong staining). The difference in expression between the different groups was tested using the chi-square statistical test.

## 3. Results

Half (*n* = 5) of the patients had the erythematotelangiectatic form, and the other half (*n* = 5) had the papulopustular form. Immunostaining was compared with six age- and sex-matched non-lesional facial skin samples. None of the control subjects had, at the time of the biopsy or in the past, rosacea.

**Figure 1:**
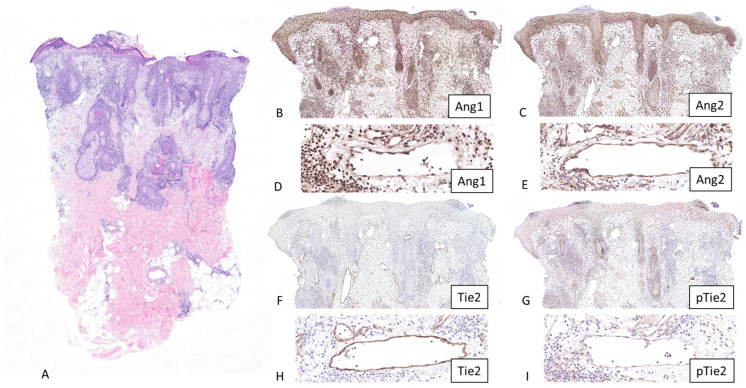
Angiopoietin 1 (Ang1), Angiopoietin 2 (Ang2), Tie2, and pTie2 stainings in rosacea skin. Skin biopsy of a rosacea patient with papulopustular lesions shows subcorneal/intraepidermal collections of neutrophils realizing pustules, ectatic dermal vessels, and perivascular and perifollicular inflammatory infiltrate of lymphocytes and histiocytes ((**A**), hematoxylin eosin staining, original magnification, 2×). Dermal vessels show an increased immunostaining for Angiopoietin 2 ((**C**), original magnification, 6.5× and (**E**), original magnification, 26×) and Tie2 ((**F**), original magnification, 6.5× and (**H**), original magnification, 26×), when compared with the immunostainings for Angiopoietin 1 ((**B**), original magnification, 6.5× and (**D**), original magnification, 26×) and pTie2 ((**G**), original magnification, 6.5× and (**I**), original magnification, 26×). The evaluation of immunostaining was performed in a blinded fashion using a scoring system of 0–3 according to the intensity of staining (0: no staining, 1+: slight staining, 2+: moderate staining, 3+: strong staining) after the examination of 3 microscopic fields per subject by a board-certified dermatopathologist (GK).

**Figure 2:**
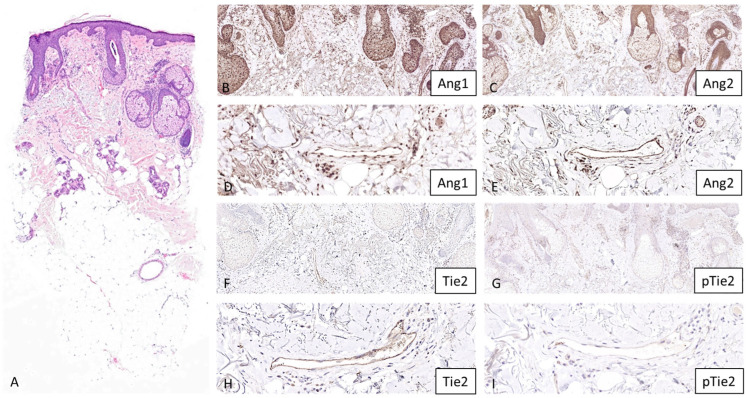
Angiopoietin 1 (Ang1), Angiopoietin 2 (Ang2), Tie2, and pTie2 stainings in rosacea skin. Skin biopsy of a rosacea patient with erythematotelangiectatic lesions shows ectatic dermal vessels, perivascular and perifollicular inflammatory infiltrate of lymphocytes and histiocytes, and intrafollicular *Demodex* mites ((**A**), hematoxylin eosin staining, original magnification, 2×). Dermal vessels show an increased immunostaining for Angiopoietin 2 ((**C**), original magnification, 6.5× and (**E**), original magnification, 26×) and Tie2 ((**F**), original magnification, 6.5× and (**H**), original magnification, 26×), when compared with the immunostainings for Angiopoietin 1 ((**B**), original magnification, 6.5× and (**D**), original magnification, 26×) and pTie2 ((**G**), original magnification, 6.5× and (**I**), original magnification, 26×). The evaluation of immunostaining was performed in a blinded fashion using a scoring system of 0–3 according to the intensity of staining (0: no staining, 1+: slight staining, 2+: moderate staining, 3+: strong staining) after the examination of 3 microscopic fields per subject by a board-certified dermatopathologist (GK).

Histological examination of papulopustular rosacea lesions showed subcorneal/intraepidermal collections of neutrophils realizing pustules, ectatic dermal vessels, and a dense perivascular and perifollicular inflammatory infiltrate of lymphocytes and histiocytes. Erythematotelangiectatic rosacea lesions revealed ectatic dermal vessels, perivascular and perifollicular inflammatory infiltrate of lymphocytes and histiocytes, and intrafollicular *Demodex* mites (Figure 1 and Figure 2). There were no signs of granulomatous or phymatous rosacea.

All markers were expressed in the dermal vessels of 100% of control and rosacea skin specimens. However, the intensity of expression differed according to the specimen and the marker (Figure 1 and Figure 2). An important background expression in the keratinocytes and the dermal cells was observed with Angiopoietin 1 staining. A similar expression pattern was also observed with Angiopoietin 2 staining, but when compared with Angiopoietin 1 expression, this was less pronounced.

**Figure 3:**
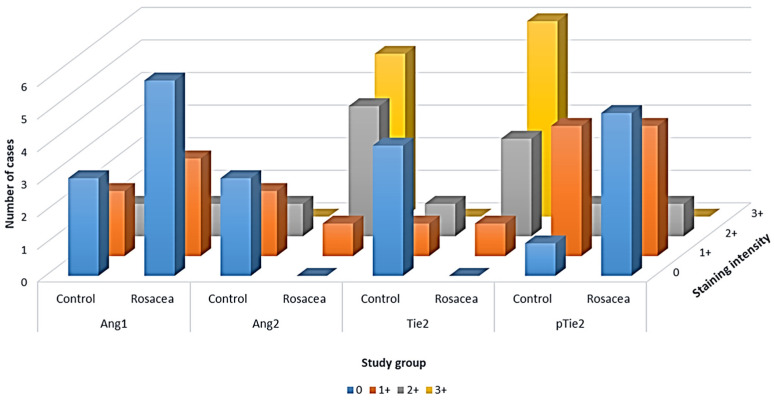
Distribution of the expression of Angiopoietin 1 (Ang1), Angiopoietin 2 (Ang2), Tie2, and pTie2 in control and rosacea skin.

In vessels, there was no difference in expression for Angiopoietin 1 between the controls and rosacea patients (Table 1, Figure 3).

A statistically significant difference (*p* < 0.05, chi-square = 94.1) was observed with Angiopoietin 2 (50% (3/6) showed no staining, 33.3% (2/6) showed grade 1 staining, and 16.7% (1/6) showed grade 2 staining in control group, and 10% (1/10) showed grade 1 staining, 40% (4/10) showed grade 2 staining, and 50% (5/10) showed grade 3 staining in rosacea group).

Similarly, a statistically significant difference (*p* < 0.05, chi-square = 120.1) was observed with Tie2 (66.7% (4/6) showed no staining, 16.65% (1/6) showed grade 1 staining, and 16.65% (1/6) showed grade 2 staining in control group, and 10% (1/10) showed grade 1 staining, 30% (3/10) showed grade 2 staining, and 60% (6/10) showed grade 3 staining in rosacea group).

There was no difference for pTie2 (16.65% (1/6) showed no staining, 66.7% (4/6) showed grade 1 staining, and 16.65% (1/6) showed grade 2 staining in control group, and 50% (5/10) showed no staining, 40% (4/10) showed grade 1 staining, and 10% (1/10) showed grade 2 staining in rosacea group) (Table 1, Figure 3). There was no difference between the erythematotelangiectatic and papulopustular forms of rosacea in terms of the results obtained.

## 4. Discussion

**Figure 4:**
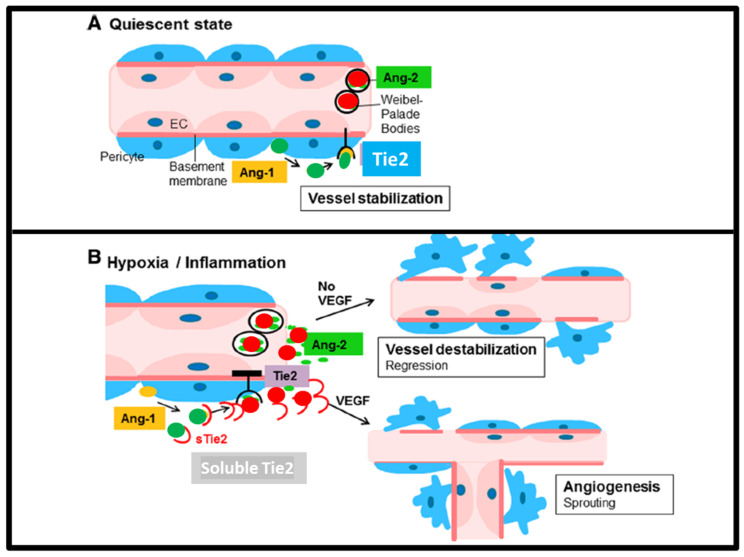
The loss of vascular quiescence WHO and HOW. Vascular quiescence: Ang1, produced by pericytes (green circles), binds its receptor Tie2 on endothelial cell membranes. This permanent activation allows vessel stabilization or quiescence (**A**). Loss of quiescence occurs when Ang2, produced by endothelial cells and stored in Weibel–Palade bodies (red circles), is released out in hypoxia or inflammation. Ang2 blocks access of Ang1 to Tie2. Inhibition of Tie2 signaling by Ang2 induces vessel destabilization. Loss of quiescence results in either apoptosis and regression of the vessel if no VEGF is available, or in neo-angiogenesis if VEGF is available. Soluble Tie2 acts as a complex modulator: Tie2 released from endothelial cell membranes binds to both Ang1 and Ang2, thus inhibiting their binding to cellular Tie receptors (**B**) [8].

This study, for the first time in human rosacea skin, shows the expression of Angiopoietin 1&2 and Tie2, the “vascular quiescence” pathway players (Figure 4).

As expected in the lesional skin of rosacea, the key keeper of vascular quiescence, Ang1 was almost not detectable, whereas its antagonist Ang2 and the Tie2 receptor were increased. This is a confirmation of our previous hypothesis that the Ang/Tie2 pathway might be a strategic target for rosacea management [7].

The angiopoietin family of angiogenic factors, including Angiopoietin 1, Angiopoietin 2, Angiopoietin 3, and Angiopoietin 4, are important for angiogenesis [9]. These factors act through the endothelial receptor tyrosine kinases Tie1 and Tie2. In contrast to the VEGF pathway, which is implicated in the initial phases of angiogenesis such as endothelial cell sprouting, the Angiopoietin–Tie system plays a role in later stages, such as vascular assembly, vascular stability and homeostasis, and endothelial quiescence [10,11,12].

**Figure 5:**
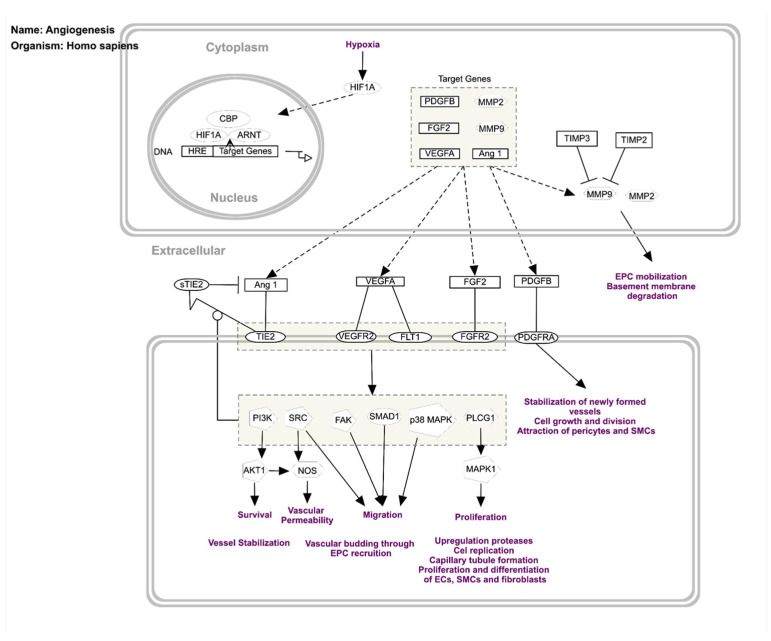
Molecules implicated in angiogenesis. This multistep biological process is mainly regulated by hypoxia. Hypoxia-inducible factor (HIF), the key transcriptional regulator of this process, induces the transcription of target genes such as Platelet-Derived Growth Factor-beta (PDGFB), Fibroblast Growth Factor 2 (FGF2), VEGFA, matrix metalloproteinase-2 (MMP2), MMP9, and Ang1. These molecules, by binding to their receptors, initiate different pathways leading to vessel formation [13].

Hypoxia plays an essential role in the regulation of angiogenesis by inducing target genes such as Platelet-Derived Growth Factor-beta (PDGFB), Fibroblast Growth Factor 2 (FGF2), VEGFA, matrix metalloproteinase-2 (MMP2), MMP9, and Angiopoietin 1 (Figure 5) [13]. Angiopoietin 1 and Angiopoietin 2 have different effects on the Tie2 receptor. Both bind to Tie2, but it is Angiopoietin 1 that is the main activator of Tie2, whereas Angiopoietin 2 has mainly an antagonist effect. However, it has been shown that in certain conditions, Angiopoietin 2 can activate Tie2 as well, exhibiting a context-dependent behavior. Angiopoietin 2 might be antagonistic on Tie2 in vascular endothelial cells but might show an agonistic effect in lymphatic vessels. Angiopoietin 2 shows a proangiogenic activity in endothelial progenitor cells via Tie2 signaling [14]. Angiopoietin 2 has been shown to act with VEGF to initiate angiogenesis. Tie1 and Tie2 receptors are transmembrane proteins. In the extracellular portion, there are Ig-like domains, fibronectin type III domains, and EGF-like domains. In the intracytoplasmic portion both receptors have tyrosine kinase domains. The kinase activity is higher in Tie2. Angiopoietin 1 and Angiopoietin 2 bind to Tie2 but not to Tie1. Upon binding, the Tie2 receptor becomes phosphorylated on several intracytoplasmic tyrosine residues, leading to the activation of downstream signaling pathways such as PI3-kinase/AKT and ERK [13]. In contrast, Tie1 does not bind directly to Angiopoietin 1 and Angiopoietin 2; however, it interacts and forms a complex with Tie2, and also becomes phosphorylated on tyrosine residues [15]. Soluble Tie2 ectodomain regulates Angiopoietin signaling by binding and blocking Angiopoietin 1 (Figure 5) [16]. Knockdown of Tie1 in mice shows that Tie1 is not essential for the AKT and ERK activation, suggesting an unclear role of Tie1 in angiopoietin signaling [17]. Tie2 knockout mice exhibit several vascular anomalies leading to early embryonic lethality [18], whereas genetic deletion of Tie1 in mice also causes embryonic lethality by vascular development abnormalities, causing a lethality in later embryonic life [19]. Venous malformations in human cases were linked to the activating mutations in the Tie2 gene, which usually occur in the kinase domain of the receptor [20].

The Angiopoietin 1&2–Tie2 signaling pathway contributes to the regulation and dysregulation of endothelial permeability during inflammation [21]. The Angiopoietin 2 and Tie2 interaction has been shown to play a role in psoriasis. Angiopoietin 2 and Tie2 are upregulated in psoriatic skin in the endothelial cells of the vessels located in the papillary dermis in the vicinity of hyperproliferative epidermis, which abundantly express VEGF. VEGF and basic FGF (bFGF) are known to induce Angiopoietin 2 expression. Successful treatment of psoriasis results in downregulation of Angiopoietin 2, suggesting that Angiopoietin 2 might be an interesting target for antipsoriatic therapies [22].

Besides VEGF and bFGF, other factors may increase the expression of Angiopoietin 2 in the skin. For example, UV irradiation has been shown to induce the release of Angiopoietin 2 from dermal vascular endothelial cells as a mechanism of UV-induced erythema characterized by inflammatory and angiogenic response, supporting the potential role of UV in the pathogenesis of rosacea [23].

Angiopoietin 2, other than VEGF, has been shown to be significantly upregulated in rosacea lesions [24]. An anti-malarial drug, Artemisinin, decreased the expression of Angiopoietin 2 and VEGFC, but not VEGFA or VEGFB, in a mouse model of rosacea induced by LL37, the proinflammatory form of cathelicidin [25], and showed a beneficial clinical effect in rosacea patients [26]. Since VEGFC is known to have a selectivity towards lymphatic vessels [27], targeting of lymphangiogenesis seems to be a possible mechanism to explain the observed positive clinical effect of Artemisinin in rosacea.

Other factors involved in the angiogenesis of rosacea lesions have been explored in past studies. Gomaa et al. reported an increased expression of VEGF, CD31, and D2-40 (podoplanin) in the lesions of non-phymatous erythematotelangiectatic and papulopustular rosacea. In this study, they pointed out that, besides angiogenesis demonstrated by the increased expression of VEGF and CD31 in the blood vessels, lymphangiogenesis, as shown by the increased numbers of D2-40-positive lymphatic vessels in rosacea skin, could also be an important factor in the pathogenesis of rosacea [28]. In another study, VEGF receptors VEGFR1 and VEGFR2 have been shown to be highly expressed in the vascular endothelium and also in inflammatory cells, including lymphocytes and plasma cells of rosacea skin samples [29]. It was shown that MMP9 expression was increased in granulomatous rosacea lesions compared with non-granulomatous rosacea lesions [30]. Abundant cytoplasmic localization has been shown in the epidermis and inflammatory cells for pS6, the phosphorylated form of the mTORC1 (mammalian target of rapamycin complex 1) downstream molecule S6 in rosacea skin [31]. Increased expression of TLR2, which activates mTORC1 signaling upon binding of LL37, has been found in the epidermis and inflammatory infiltrate of erythematotelangiectatic and granulomatous rosacea [4,32].

Our results show that Ang2 levels are increased in lesional rosacea skin, possibly leading to disruption of the stabilization of Ang1/Tie2 signaling. By blocking this signaling, Ang2 highly likely leads to the detachment of supporting pericytes and loosening of vascular cell contacts, making the vessels unstable and leaky. This vascular sensitization would make the blood vessels more responsive to VEGF, another key proangiogenic factor highly expressed in rosacea. Inflammatory mediators, such as LL-37 and various cytokines released in rosacea, would further contribute to this process by promoting angiogenesis and inflammation, which in turn can lead to more Ang2 expression, perpetuating a vicious cycle of disease progression. However, the elevated expression of Ang2 and Tie2 in the endothelial cells of dermal vessels in rosacea skin with no change in pTie2, the activated form of Tie2, may represent a secondary phenomenon or a downstream final consequence of the disease rather than a primary trigger or a pathogenetic mechanism.

## 5. Conclusions

Our study indicates that the Angiopoietin 1&2/Tie2 signaling pathway is likely a critical component in the pathophysiology of rosacea and a strategic target for rosacea management, specifically through increasing expression of Angiopoietin 2. We acknowledge the limitations of our study due to the small sample size and lack of inter-observer validation, and larger series will be required to validate these observations.

## Figures and Tables

**Table 1:** Expression of different vascular markers in dermal vessels in study groups.

	Group			
	Control Skin		Rosacea Skin	
Marker	Count	%	Count	%
**Angiopoietin 1**				
0	3	50	6	60
1+	2	33.3	3	30
2+	1	16.7	1	10
3+	0	0	0	0
Total	6	100	10	100
**Angiopoietin 2**				
0	3	50	0	0
1+	2	33.3	1	10
2+	1	16.7	4	40
3+	0	0	5	50
Total	6	100	10	100
**Tie2**				
0	4	66.7	0	0
1+	1	16.65	1	10
2+	1	16.65	3	30
3+	0	0	6	60
Total	6	100	10	100
**pTie2**				
0	1	16.65	5	50
1+	4	66.7	4	40
2+	1	16.65	1	10
3+	0	0	0	0
Total	6	100	10	100

0: no staining, 1+: slight staining, 2+: moderate staining, 3+: strong staining.

## Data Availability

The data presented in this study are available on request from the corresponding author.
